# Functional Improvement at One Year in Fibrotic Interstitial Lung Diseases—Prognostic Value of Baseline Biomarkers and Anti-Inflammatory Therapies

**DOI:** 10.3390/diagnostics14141544

**Published:** 2024-07-17

**Authors:** Guangyu Shao, Paul Thöne, Bernhard Kaiser, Bernd Lamprecht, David Lang

**Affiliations:** 1 Kepler University Hospital, 4020 Linz, Austriadavid.lang@kepleruniklinikum.at (D.L.); 2 Faculty of Medicine, Johannes Kepler University, 4040 Linz, Austria

**Keywords:** interstitial lung disease, biomarker, prognostication

## Abstract

Background: The clinical spectrum of fibrotic interstitial lung diseases (ILDs) is highly heterogeneous. We aimed to evaluate the prognostic value of widely available baseline biomarkers for the improvement of lung function in patients with fibrotic ILDs. Methods: This registry-based study included 142 patients with fibrotic ILDs as defined by the presence of reticulation, traction bronchiectasis or honeycombing on initial high-resolution computed tomography (HRCT). Functional improvement at 1 year was defined as a relative increase of 5% in forced vital capacity (FVC) or of 10% in diffusion capacity for carbon monoxide (DLCO). The prognostic value of baseline biomarkers was evaluated for all patients and the subgroup with anti-inflammatory treatment. Results: At one year, 44 patients showed improvement while 73 showed disease progression. Multivariate analyses found prognostic significance for age < 60 years (OR 5.4; 95%CI 1.9–15.4; *p* = 0.002), lactate dehydrogenase (LDH) >250 U/L (OR 2.5; 95%CI 1.1–5.8; *p* = 0.043) and blood monocyte count < 0.8 G/L (OR 3.5; 95%CI 1.1–11.3; *p* = 0.034). In 84 patients undergoing anti-inflammatory treatment, multivariate analysis revealed age < 60 years (OR 8.5 (95%CI 2.1–33.4; *p* = 0.002) as the only significant variable. Conclusion: Younger age, a higher LDH and lower blood monocyte count predicted functional improvement in fibrotic ILD patients, while in those treated with anti-inflammatory drugs, only age had significant implications.

## 1. Introduction

Interstitial lung diseases (ILDs) encompass a wide spectrum of pulmonary disorders characterized by inflammation and fibrosis of the pulmonary alveolar, vascular and interstitial domains [1]. Clinical outcomes vary considerably depending on the specific ILD subtypes and individual patient characteristics. ILDs with an assumed inflammatory etiology are more likely to response to immunosuppressive treatment, resulting in improved survival outcomes [2,3,4,5]. In contrast, fibrotic ILDs carry a higher risk of progressive and irreversible loss of lung function, ultimately leading to respiratory failure and death [6]. Recently, the official ATS/ERS/JRS/ALAT guideline introduced the term “progressive pulmonary fibrosis” (PPF) to describe the progressive subgroup of fibrotic ILDs other than idiopathic pulmonary fibrosis (IPF) [7]. This novel classification emphasizes the fact that a heterogenous group of conditions may resemble the disease course of IPF [8]. Consequently, the available literature supports early detection of both IPF and PPF, as antifibrotic agents including pirfenidone or nintedanib can reduce loss of lung function, leading to a more favorable outcome [9]. However, only a variable fraction of 18 to 32% of non-IPF ILDs will progress significantly, making the identification of PPF challenging [10]. Moreover, evidence for the use of antifibrotic therapy in patients presenting with fibrotic non-IPF ILD at first diagnosis is limited [11]. In fact, in those patients, immunosuppression is still the most common initial therapeutic approach [3].

Over the last several years, much research effort has been dedicated to identifying predictive biomarkers that enable personalized risk assessment in ILDs. Significant impact on disease course was observed for age, sex, body mass index, specific gene variants like MUC5B, blood monocyte count and pulmonary function tests (PFTs) [12]. Recent research shows promising results for proteomics-based molecular biomarkers, but these are not commonly available in clinical practice yet [13,14]. In addition, presence and extent of fibrosis on HRCT have been identified as highly significant risk factors for disease progression in various ILD subtypes [10,15]. However, not all patients presenting with fibrotic ILDs will experience progression, but a significant proportion may even demonstrate functional improvement over time, reflecting the heterogeneous nature of the disease, as shown in SSC-ILD [16]. Limited evidence exists regarding baseline biomarkers that could predict such improvement in lung function despite fibrotic CT patterns. Moreover, considering that the majority of non-IPF ILD patients receives immunomodulatory or immunosuppressant drugs for initial treatment, there is also a lack of data on the impact of this therapeutic approach both in terms of beneficial effects but also concerning the risk of developing PPF.

In the present study, our objective was to examine the correlation between widely available baseline biomarkers and improvement of lung function after one year in patients with fibrotic ILDs of various causes. Furthermore, we assessed the possible impact of anti-inflammatory therapies in the study cohort.

## 2. Materials and Methods

This retrospective study is conducted using data obtained from patients included in an institutional ILD registry at Kepler University Hospital in Linz, Austria, between 2017 and 2021. It was performed in accordance with the Declaration of Helsinki and received approval as well as a reassessment on a yearly basis by the ethics committee of the Medical Faculty of Linz (study number I-26-17). Furthermore, the study was conducted in adherence to the Strengthening the Reporting of Observational Studies in Epidemiology (STROBE) guidelines for reporting observational studies [17].

All patients included for the evaluation provided written informed consent, which was discussed by a multidisciplinary team in the local ILD board. Before that, a standardized ILD examination was performed, including physical examination, HRCT imaging, laboratory analyses and PFTs including spirometry, body plethysmography and measurement of diffusion capacity (JAEGER Master Screen PFT/Body/Diffusion, CareFusion, San Diego, CA, USA). To be eligible for this study, all patients were required to have at least one of the typical CT hallmarks of pulmonary fibrosis, including reticular abnormalities, traction bronchiectasis, or honeycombing on initial HRCT. One-year survival data as well as at least one follow-up PFT assessment after initial examination had to be available. Treatment with systemic corticosteroids, immunomodulatory or immunosuppressant drugs was categorized as “anti-inflammatory” when its duration was at least 6 weeks, and it had been primarily prescribed for the management of ILDs and not for treating other conditions.

HRCT chest images were obtained in prone position whenever clinically feasible, following protocols proposed by the relevant guidelines [18]. The presence of parenchymal nodules, reticulation, honeycombing, consolidations, ground glass opacities, emphysema, mosaic attenuation and traction bronchiectasis was assessed by a specialist ILD radiologist during the respective ILD board session. For semi-quantitative analysis of the ILD findings, the lungs were divided into an upper-, middle- and lower-lung area, as defined by a third of the largest craniocaudal diameter in the sagittal reconstructions. This approach resulted in a simple scoring system from one to six, as described in our previous publications [15,19].

Blood samples were analyzed using a Sysmex^®^ XN-3000 hematology analyzer (Sysmex Europe GmbH, Norderstedt, Germany) for cell counts and a Cobas^®^ 8000 modular analyzer (Roche Diagnostics International AG, Rotkreuz, Switzerland) for C-reactive protein (CRP), lactate dehydrogenase (LDH) and rheumatoid factor measurement. Autoimmune serology testing was performed using a EuroPatternMicroscope^®^, a Dynex^®^, and a EuroBlotOne^®^ platform by Euroimmun (EUROIMMUN Medizinische Labordiagnostik AG, Lübeck, Germany) for anti-nuclear (ANA), anti-neutrophil cytoplasmatic (ANCA) and other disease-specific antibodies.

Pulmonary function tests including spirometry, body plethysmography and measurement of diffusion capacity (JAEGER MasterScreen PFT/Body/Diffusion^®^, CareFusion, San Diego, United States of America) were performed to assess forced vital capacity (FVC, L/% predicted), forced expiratory volume in 1 s (FEV1, % predicted), FEV1/FVC ratio and diffusion capacity for carbon monoxide (DLCO, single breath method, mmol/(min × kPa)/%predicted). Normal values for spirometry were based on the GLI-2012 equations and those for body plethysmography and diffusion capacity on the 1993 ERS/ECCS regressions.

**Figure 1:**
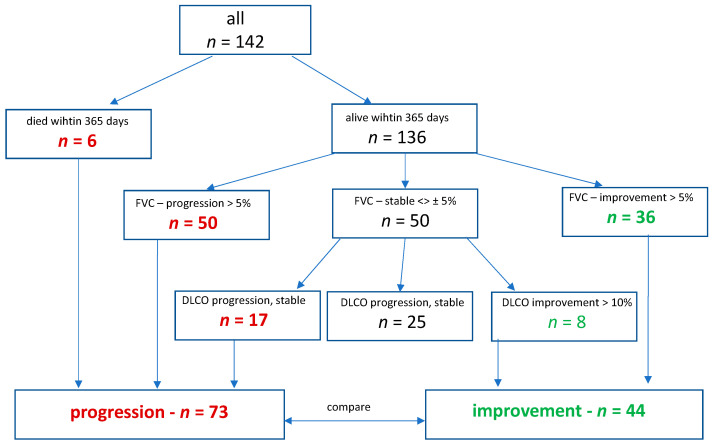
Derivation of the study cohorts. FVC, forced vital capacity; DLCO, diffusion capacity for carbon monoxide.

Functional improvement was defined as a relative increase of either >5% in FVC (% predicted) or, in case of stable FVC, a relative increase of 10% in DLCO within the first year after primary evaluation, regardless of the timing of the event during that time span. Disease progression was defined by a composite endpoint, including a relative decrease of 5% in FVC, 10% in DLCO, lung transplant or death with the first year. After a follow-up period of one year, 2 cohorts were formed, with 44 patients showing functional improvement and 73 patients showing disease progression (Figure 1).

All statistical analyses were performed using R (version 4.3.0). Descriptive analyses included calculations of measures such as the mean, standard error and proportions. Statistical tests, both parametric and non-parametric, were employed depending on the respective hypotheses and normal distribution of the data. These tests included the *t*-test, Kruskal–Wallis test, Mann–Whitney U test, Spearman’s rank correlation test, Chi-squared test, and Fisher’s exact test.

In preparation for the multivariate analysis, continuous variables such as age, LDH and monocytes were dichotomized into two categories. This dichotomization was based on a third variable, the presence or absence of functional improvement, with the aim of finding a cutoff value with high selectivity. For instance, the cutoff age of 60 years was identified as the optimal threshold for predicting improvement. Although dichotomization can lead to a loss of information, binary variables are often easier to interpret and more practical for implementation in medical practice.

To determine the optimal cutoff value, the CUTPOINTR-package in R (R: A language and Environment for Statistical Computing. R Foundation for Statistical Computing, Vienna, Austria; Version 3.6.0) was utilized. Significant predictors of improvement were identified using logistic regression models. The odds ratios presented in the analysis indicate the likelihood of improvement versus deterioration of lung function.

## 3. Results

Out of 209 patients enrolled in the ILD registry between 2017 and 2022, 142 patients were eligible for the analysis. Most patients had been diagnosed with connective tissue disease-ILD (CTD-ILD; 22.5%), followed by idiopathic nonspecific interstitial pneumonia (iNSIP; 21.1%), idiopathic pulmonary fibrosis (IPF; 15.5%), interstitial pneumonia with autoimmune features (IPAF; 13.4%) and chronic hypersensitivity pneumonitis (CHP; 7.8%). During the first year, six patients died, fifty experienced significant worsening in FVC and thirty-six patients showed improvement. Of the remaining 50 patients with stable FVC, 17 experienced significant worsening of DLCO at one year, while 8 showed improvement. Thus, 2 cohorts were established, consisting of 73 patients with disease progression and 44 patients with functional improvement.

### 3.1. Primary Outcome

Respective baseline characteristics, PFTs, BAL fluid, peripheral blood and HRCT findings for all patients, as well as separately for the progressive, stable and improved cohorts are shown in Table 1. Significant association with functional improvement could be observed for younger age (*p* = 0.023), lower FVC (*p* = 0.017) and FEV-1% of predicted (*p* = 0.018), lower peripheral blood monocyte count (*p* = 0.046), elevated proportions of lymphocytes in BAL fluid (*p* = 0.038), as well as for higher extent of GGO (*p* = 0.047) and less traction bronchiectasis (*p* = 0.019). Also, ILD-board diagnosis had significant implications on the course of disease (*p* = 0.024).

**Figure 2:**
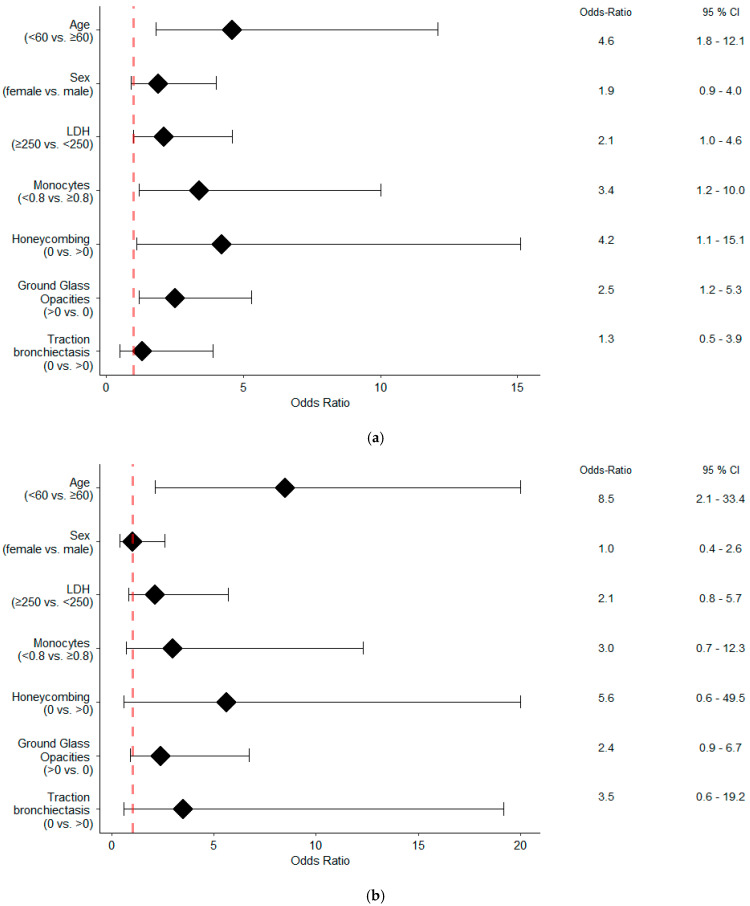
Forest-plots illustrating univariate analyses of all patients (**a**) and the anti-inflammatory subgroup (**b**). The odds ratios displayed represent the likelihood of functional improvement as compared to deterioration. LDH, lactate dehydrogenase.

Univariate analysis demonstrated a significant association with functional improvement at one year for age (<60 vs. ≥60; OR 4.6; 95%CI 1.8–12.1; *p* = 0.002), honeycombing score (0 vs. ≥0; OR 4.2; 95%CI 1.1–15.1; *p* = 0.031), GGO score (≥0 vs.0; OR 2.5; 95%CI 1.2–5.3; *p* = 0.021), DLCO% of predicted (<43% vs. ≥43%; OR 2.9; 95%CI 1.3–6.7; *p* = 0.010) and FVC % of predicted (<95% vs. ≥95%; OR 4.6; 95%CI 1.5–14.4; *p* = 0.009), as shown by Table 2 and Figure 2a. In multivariate analysis excluding FVC% and DLCO% of predicted, functional improvement at one year was associated with age (<60 vs. ≥60; OR 5.4; 95%CI 1.9–15.4; *p* = 0.002), LDH (>250 U/L vs. ≤250 U/L; OR 2.5; 95%CI 1.1–5.8; *p* = 0.043) and monocyte count (<0.8 G/L vs. ≥0.8 G/L; OR 3.5; 95%CI 1.1–11.3; *p* = 0.034), as shown in Table 2.

Relative BAL lymphocyte counts exceeding the calculated cutoff at 12% had significant implications on functional improvement in a univariate model of all patients (OR 3.52; 95%CI 1.27–9.75; *p* = 0.016). However, this association was not observed in patients treated with anti-inflammatory therapies (OR 2.08; 95%CI 0.65–6.71; *p* = 0.22). In both groups, there was no significant effect of BAL lymphocyte counts in multivariate analyses, likely due to the limited number of patients with available BAL data. Consequently, this variable was excluded from the final evaluation.

Functional improvement was observed in 66.7% of the patients aged <60 as compared to only 30.1% of the patients aged ≥60. While 48.3% of patients with blood monocyte count <0.8 G/L experienced an improvement of lung function, only 18.5% of patients had a monocyte count >0.8 G/L. Similarly, 44.7% of patients with LDH >250 U/L had functional improvement, in contrast to 30.3% of patients with LDH <250 U/L.

### 3.2. Secondary Outcome

Out of the total of 142 patients, 84 had received anti-inflammatory treatment as previously defined within the first year. Among them, 38 patients (45%) experienced disease progression, whereas 31 (37%) improved, and 15 patients (18%) remained stable.

Respective baseline characteristics, PFTs, peripheral blood, BAL fluid and HRCT findings for all patients undergoing anti-inflammatory treatment, as well as separately for the progression, stable and improvement cohorts are shown in Table 3. Significant associations with functional improvement could be observed for lower FVC% of predicted (*p* = 0.008), FEV-1% of predicted (*p* = 0.018) and lower traction bronchiectasis (*p*= 0.043).

Univariate analysis demonstrated a significant association with function improvement at one year for age (<60 vs. ≥60; OR 8.5; 95%CI 2.1–33.4; *p* = 0.002), DLCO% of predicted (<43% vs. ≥43%; OR 3.6; 95%CI 1.2–10.8; *p* = 0.019) and FVC% of predicted (<95% vs. ≥95%; OR 9.3; 95%CI 1.1–78.2; *p* = 0.040), as shown by Table 2 and Figure 2b. Of interest, the multivariate analysis excluding FVC% and DLCO% of predicted identified younger age (<60 vs. ≥60; OR 8.5; 95%CI 2.1–33.4; *p* = 0.002) as the only significant variable associated with functional improvement.

The baseline and one-year follow-up PFTs including FVC, FEV1 and DLCO were analyzed for all patients and for those undergoing anti-inflammatory treatment across the progression, stable and improvement cohorts. Detailed results are provided in the Supplementary Materials (Tables S1 and S2, Figures S1–S6).

## 4. Discussion

The results of this retrospective, registry-based evaluation suggest that younger age, higher LDH level and lower peripheral blood monocyte count were associated with an improvement of lung function within one year in fibrotic ILDs, irrespective of the underlying diagnosis or treatment. Among patients undergoing anti-inflammatory treatment, only younger age showed significant implications.

These findings are in accordance with the existing prognostic models for fibrotic ILDs, with the gender–age–physiology (GAP) score being the most commonly used [12,20]. In this staging system derived from IPF patients, older age was a significant predictor of mortality. Our observation of an association of younger age with functional improvement may be attributed to the higher prevalence of PPF and IPF in older individuals, as reported in several epidemiological studies [21,22,23,24]. In one study conducted by Hopkins et al., the prevalence for IPF increased twentyfold from the age group 50–59 to the >90 age group for both genders [23]. In contrast, younger patients tend to experience ILDs with autoimmune pathogenesis that carry a more favorable prognosis than IPF [25,26]. In line with this, we found LDH, a well-established indicator of inflammation, to be significantly higher in the subgroup with functional improvement. Conversely, an elevation of LDH was found to be associated with a progression of ILDs in connective tissue diseases as well as in other autoimmune diseases, including Sjögren’s and anti-synthetase syndrome [27,28,29]. In addition to a possible role of LDH as a marker of generally increased disease activity leading to worse prognosis, these conflicting results may be because approximately half of our study population received anti-inflammatory treatment. In the multivariate analysis of this subgroup, the protective effect of elevated LDH diminished, possibly reflecting its potential to serve as an indicator of inflammatory disease activity and also treatment response.

Numerous studies have reported the prognostic significance of monocytes for disease progression in IPF and other fibrotic ILD subtypes [19,30,31]. On the cellular level, monocytes are recruited to the lung following tissue injury, where they differentiate into profibrotic alveolar macrophages, contributing to pulmonary fibrosis [32,33]. In contrast, a depletion of monocytes was shown to reduce the extent of fibrosis in a mouse model, and IPF patients with normal monocyte counts were reported to have a significantly improved survival [34]. Consistent with this pathophysiological model, our findings suggest that a lower level of circulating monocytes is predictive for the improvement of lung function in fibrotic ILDs. Interestingly, the prognostic value of a lower monocyte count diminished in the anti-inflammatory therapy subgroup. Results of previous studies reported alterations in monocyte count and activity following the use of immunosuppressants, such as glucocorticoids or methotrexate [35,36]. However, the influence of these treatment effects on our findings is unlikely, as the monocyte count was assessed prior to receiving any ILD-specific treatment in the majority of the patients. Thus, in our study cohort, elevated monocyte counts probably indicated the risk of progressive fibrosis in untreated ILDs, whereas lower levels of monocytes were associated with improved lung function. However, in fibrotic ILD patients receiving anti-inflammatory treatment, baseline monocyte count showed no predictive value in terms of functional improvement.

To our knowledge, this is the first study assessing prognostic biomarkers to predict functional improvement in fibrotic ILDs. The growing knowledge of risk factors associated with IPF and PPF has led to an improved recognition of patients who are likely to progress and can benefit from antifibrotic therapy. However, a significant number of fibrotic ILD patients is still treated with immunosuppressants either due to an assumed underlying inflammation-driven disease or for not meeting criteria for diagnosing IPF or disease progression, thereby allowing the prescription of antifibrotics [3]. In the appropriate clinical context, the findings of the present study support a trial of immunosuppressive treatment in patients with younger age, an elevated LDH level and lower monocyte count, even despite fibrotic appearance on HRCT and irrespective of the underlying diagnosis. On the other hand, patients lacking these characteristics have a high risk of progression and should be treated with antifibrotic agents with a low threshold when appropriate and possible. If they still receive immunosuppressive agents, they should be monitored for progression very closely.

Limitations of this study include its retrospective, single-center design, potentially causing bias and restricting the generalizability of the findings to a broader population. Due to the registry-based and retrospective methodology, no formal sample size calculation was performed, which poses another possible limitation to the presented data. In addition, the study population was heterogeneous, consisting of patients with different fibrotic ILD subtypes and treatment regimens. However, it represents the clinical practice of a university hospital ILD clinic with an emphasis on patients with autoimmune diseases. Our analyses concerning patients treated with anti-inflammatory agents are limited by the lack of a standard treatment, leading to the use of a multitude of treatment options for various indications. The definition of “anti-inflammatory” treatment used in our study represents a minimal consensus primarily defined to enable statistical analyses. However, as therapeutic decisions were solely based on expert knowledge and clinical presentation of the patient, they may not be generalizable to other populations and differ from other centers. A major clinical advantage of the prognostic biomarkers identified in the current analysis is that they can be easily obtained in a minimally invasive and inexpensive fashion as part of the primary ILD evaluation. While age is readily available through medical records, LDH and monocyte count are typically included in routine blood tests. Although these biomarkers are unspecific when used alone, they may offer clinicians guidance in management decisions for fibrotic ILD patients, especially when several biomarkers are applied together in the context of the respective clinical data. In addition, they could allow for a decision support independent of the underlying ILD diagnosis and without having to await progression before antifibrotic therapy can be initiated.

## 5. Conclusions

In summary, we conclude that younger age, higher LDH levels and lower monocyte counts predict functional improvement in fibrotic ILDs, whereas in patients treated with anti-inflammatory drugs, only younger age prevailed as a significant predictor. Further studies with a prospective design, larger sample size, defined patient subgroups and standardized treatment are required to investigate the impact of such biomarkers more accurately.

## Figures and Tables

**Table 1:** Baseline patient, treatment and PFT characteristics, baseline peripheral blood and BAL markers and baseline computed tomography scores in all patients according to disease course. SE, standard error; FVC, forced vital capacity; FEV-1, forced expiratory volume in 1 s; DLCO, diffusion capacity for carbon monoxide; BAL, bronchoalveolar lavage; CHP, chronic hypersensitivity pneumonitis; CTD-ILD, connective tissue disease–interstitial lung disease; iNSIP, idiopathic nonspecific interstitial pneumonia; iPAF, interstitial pneumonia with autoimmune features; IPF, idiopathic pulmonary fibrosis.

	All Patients (*n* = 142)				
Variable	All Patients (*n* = 142)	Progression at 1 Year (*n* = 73)	Stable at 1 Year (*n* = 25)	Improvement at 1 Year (*n* = 44)	*p*-Value
Baseline characteristics					
Mean age (SE)	67.0 (1.1)	70.4 (1.0)	64.8 (2.5)	62.4 (2.4)	0.023
Age ≥70 years (%)	47.2	54.8	36.0	40.9	0.162
Female sex (%)	36.6	32.9	28.0	47.7	0.167
Treatment characteristics (%)					
Anti-inflammatory	52.1	41.1	52.0	70.5	0.066
Antifibrotic	12.0	15.1	12.0	6.8	
Anti-inflammatory and antifibrotic	7.0	11.0	8.0	0.0	
No ILD-specific therapy	28.9	32.0	28.0	22.7	
Any anti-inflammatory treatment	59.1	52.1	60.0	70.5	0.146
Any antifibrotic treatment	19.0	26.1	20.0	6.8	0.037
Pulmonary functions tests; mean (SE)					
FVC (% pred.)	81.3 (1.5)	84.3 (2.1)	84.6 (3.9)	74.5 (2.6)	0.017
FEV1 (% pred.)	82.8 (1.6)	86.6 (2.1)	84.4 (4.1)	75.9 (2.5)	0.018
DLCO (% pred.)	55.2 (1.5)	57.1 (2.0)	58.2 (3.4)	50.3 (2.6)	0.104
Peripheral blood biomarkers; mean (SE)					
Absolute leukocyte count (G/L)	8.8 (0.3)	8.6 (0.4)	8.8 (1.0)	9.0 (0.4)	0.442
Neutrophil-to-lymphocyte ratio	5.3 (0.8)	4.7 (0.9)	6.5 (2.9)	5.8 (1.0)	0.164
Absolute monocyte count (G/L)	0.6 (0.1)	0.6 (0.1)	0.5 (0.1)	0.6 (0.1)	0.046
Absolute eosinophil count (G/L)	0.2 (0.1)	0.2 (0.1)	0.1 (0.0)	0.2 (0.1)	0.397
C-reactive protein (mg/dL)	1.2 (0.2)	0.9 (0.2)	1.1 (0.5)	1.8 (0.4)	0.285
Lactate dehydrogenase (U/L)	248.3	241.4 (9.8)	224.8 (12.1)	272.5 (15.7)	0.095
Bronchoalveolar lavage; mean (SE) *n* = 81					
BAL—Macrophage fraction	57.7 (3.1)	61.2 (4.2)	57.5 (8.3)	52.9 (5.3)	0.484
BAL—Lymphocyte fraction	17.9 (2.2)	14.7 (2.4)	9.3 (2.8)	26.5 (5.0)	0.038
BAL—Neutrophile fraction	15.1 (2.2)	17.5 (3.5)	18.9 (7.3)	10.1 (1.9)	0.592
BAL—Eosinophile fraction	4.1 (0.9)	3.8 (1.4)	5.1 (1.8)	4.1 (1.4)	0.631
Computed tomography finding scores; median (range), mean (SE)					
Reticular abnormalities	6 (0–6) 4.9 (1.6)	6 (0–6) 5.0 (1.6)	6 (0–6) 4.8 (1.9)	6 (1–6) 4.9 (1.5)	0.807
Honeycombing	0 (0–6) 0.6 (1.5)	0 (0–6) 0.9 (1.9)	0 (0–6) 0.6 (1.5)	0 (0–3) 0.2 (0.6)	0.063
Ground glass opacities	0 (0–6) 1.7 (2.3)	0 (0–6) 1.4 (2.2)	0 (0–6) 1.6 (2.4)	2 (0–6) 2.4 (2.5)	0.047
Emphysema	0 (0–6) 0.6 (1.3)	0 (0–6) 0.5 (1.2)	0 (0–6) 0.7 (1.5)	0 (0–6) 0.5 (1.4)	0.534
Traction bronchiectasis	2 (0–6) 2.8 (1.9)	2 (0–6) 3.0 (1.9)	2 (0–6) 1.8 (1.5)	2 (0–6) 3.0 (2.1)	0.019
Diagnosis (%)					
CHP	7.8	5.5	12.0	9.1	0.024
CTD-ILD	22.5	17.8	16.0	34.1	
iNSIP	21.1	27.4	24.0	9.1	
iPAF	13.4	15.1	20.0	6.8	
IPF	15.5	20.6	16.0	6.8	

**Table 2:** Results of the logistic regression for all patients (improvement *n* = 44 vs. progression *n* = 73) and for the anti-inflammatory subgroup (improvement *n* = 31 vs. progression = 38). The odds ratios displayed in the table represent the likelihood for functional improvement compared to deterioration. CI, confidence interval; LDH, lactate dehydrogenase; FVC, forced vital capacity; DLCO, diffusion capacity for carbon monoxide.

MV Analysis Dependent Variable: Improvement	Univariate				Multivariate			
	All Patients		Anti-Inflammatory		All Patients		Anti-Inflammatory	
Independent Variables	Odds Ratio (95% CI)	*p*-Value	Odds Ratio (95% CI)	*p*-Value	Odds Ratio (95% CI)	*p*-Value	Odds Ratio (95% CI)	*p*-Value
Age (<60 vs. ≥60)	4.6 (1.8–12.1)	0.002	8.5 (2.1–33.4)	0.002	5.4 (1.9–15.4)	0.002	8.5 (2.1–33.4)	0.002
Sex (female vs. male)	1.9 (0.9–4.0)	0.115	1.0 (0.4–2.6)	0.972				
LDH (≥250 vs. <250)	2.1 (1.0–4.6)	0.066	2.1 (0.8–5.7)	0.149	2.5 (1.0–5.8)	0.043		
Monocytes (<0.8 vs. ≥0.8)	3.4 (1.2–10.0)	0.022	3.0 (0.7–12.3)	0.126	3.5 (1.1–11.3)	0.034		
Honeycombing (0 vs. >0)	4.2 (1.1–15.1)	0.031	5.6 (0.6–49.5)	0.12				
Ground Glass Opacities (>0 vs. 0)	2.5 (1.2–5.3)	0.021	2.4 (0.9–6.7)	0.081				
Traction bronchiectasis (0 vs. >0)	1.3 (0.5–3.9)	0.586	3.5 (0.6–19.2)	0.156				
DLCO %-predicted (base) (<43% vs. ≥43%)	2.9 (1.3–6.7)	0.01	3.6 (1.2–10.8)	0.019	not included		not included	
FVC%-predicted (base) (<95% vs. ≥95%)	4.6 (1.5–14.4)	0.009	9.3 (1.1–78.2)	0.04	not included		not included	

**Table 3:** Baseline patient, treatment and PFT characteristics, baseline peripheral blood and BAL markers and baseline computed tomography scores in the anti-inflammatory treatment subgroup according to disease course. SE, standard error; FVC, forced vital capacity; FEV1, forced expiratory volume in 1 s; DLCO, diffusion capacity for carbon monoxide; BAL, bronchoalveolar lavage; CHP, chronic hypersensitivity pneumonitis; CTD-ILD, connective tissue disease–interstitial lung disease; iNSIP, idiopathic nonspecific interstitial pneumonia; iPAF, interstitial pneumonia with autoimmune features; IPF, idiopathic pulmonary fibrosis.

	Patients with Anti-Inflammatory Treatment (*n* = 84)				
Variable	All Patients (*n* = 84)	Progression at 1 Year (*n* = 38)	Stable at 1 Year (*n* = 15)	Improvement at 1 Year (*n* = 31)	*p*-Value
Baseline characteristics					
Mean age (SE)	66.2 (1.5)	70.2 (1.4)	66.8 (2.9)	61.1 (3.1)	0.122
Age ≥70 years (%)	40 (47.6)	22 (57.9)	6 (40.0)	12 (38.7)	0.229
Female sex (%)	35 (41.7)	17 (44.7)	4 (26.7)	14 (45.2	0.429
Pulmonary functions tests; mean (SE)					
FVC (% pred.)	80.3 (2.1)	82.9 (3.0)	89.4 (4.8)	72.7 (3.0)	0.008
FEV1 (% pred.)	81.9 (2.0)	84.9 (2.9)	89.3 (4.4)	74.8 (3.0)	0.018
DLCO (% pred.)	52.9 (1.8)	53.9 (2.1)	58.9 (5.4)	48.7 (3.1)	0.183
Treatment characteristics (%)					
Additional antifibrotic treatment	10 (11,9%)	8 (80%)	2 (20%)	0 (0%)	0,027
Peripheral blood biomarkers; mean (SE)					
Absolute leukocyte count (G/L)	8.9 (0.3)	8.7 (0.6)	8.5 (0.9)	9.2 (0.5)	0.379
Neutrophil-to-lymphocyte ratio	5.7 (0.9)	5.7 (1.7)	4.1 (1.1)	6.5 (1.1)	0.170
Absolute monocyte count (G/L)	0.6 (0.1)	0.6 (0.1)	0.6 (0.1)	0.5 (0.1)	0.638
Absolute eosinophil count (G/L)	0.2 (0.1)	0.2 (0.1)	0.1 (0.0)	0.2 (0.1)	0.662
C-reactive protein (mg/dL)	1.2 (0.2)	0.7 (0.2)	0.7 (0.2)	2.1 (0.6)	0.308
Lactate dehydrogenase (U/L)	259.7 (11.1)	253.1 (16.2)	218.9 (12.7)	288.6 (21.0)	0.078
Bronchoalveolar lavage; mean (SE) *n* = 81					
BAL—Macrophage fraction	54.9 (3.5)	58.7 (4.9)	59.7 (9.9)	49.0 (5.7)	0.429
BAL—Lymphocyte fraction	22.1 (2.9)	19.3 (3.3)	11.9 (3.7)	28.9 (5.7)	0.215
BAL—Neutrophile fraction	12.8 (1.9)	13.0 (3.1)	17.4 (6.6)	10.8 (2.2)	0.551
BAL—Eosinophile fraction	4.7 (1.2)	4.6 (2.1)	5.6 (2.4)	4.5 (1.6)	0.647
Computed tomography finding scores; median (range), mean (SE)					
Reticular abnormalities	6 (0–6) 4.8 (1.7)	6 (0–6) 4.6 (1.8)	6 (2–6) 4.9 (1.7)	6 (1–6) 4.9 (1.5)	0.740
Honeycombing	0 (0–6) 0.3 (1.1)	0 (0–6) 0.6 (1.5)	0 (0–2) 0.1 (0.5)	0 (0–2) 0.1 (0.4)	0.179
Ground glass opacities	1.5 (0–6) 2.2 (2.5)	0.5 (0–6) 1.9 (2.4)	0 (0–6) 1.7 (2.4)	2 (0–6) 2.9 (2.5)	0.118
Emphysema	0 (0–6) 0.5 (1.2)	0 (0–6) 0.6 (1.2)	0 (0–6) 0.8 (1.8)	0 (0–4) 0.2 (0.7)	0.151
Traction bronchiectasis	2 (0–6) 2.9 (2.0)	2 (0–6) 3.1 (1.8)	2 (0–6) 1.7 (1.7)	2 (0–6) 3.2 (2.2)	0.043
Diagnosis (%)					
CHP	10.7	10.5	6.7	12.9	0.232
CTD-ILD	23.8	21.1	20.0	29.0	
iNSIP	19.1	23.7	33.3	6.5	
iPAF	13.1	15.8	13.3	9.7	
IPF	4.8	5.3	13.3	0.0	

## Data Availability

As mandated by the ethics committee of Medical Faculty Linz, publication or dissemination of any possibly identifiable patient data from the Kepler University Hospital ILD registry is strictly prohibited. The dataset used for the analyses contains very detailed and thus possibly identifiable patient data, so that a publication of the database is not possible. However, upon reasonable request to the authors and if permitted by the Ethics Committee of the Medical Faculty of Linz in an amendment to the study protocol, anonymized data may under certain circumstances be shared.

## Supplementary Materials

The following supporting information can be downloaded at: https://www.mdpi.com/article/10.3390/diagnostics14141544/s1, Table S1. Baseline and one-year follow-up pulmonary function tests (PFTs) of all patients. FVC, Forced Vital Capacity; FEV1, forced expiratory volume in 1 s; DLCS, diffusion capacity for carbon monoxide. Figure S1. Baseline and one-year follow-up pulmonary function tests (PFTs) of all patients. FVC, Forced Vital Capacity. Figure S2. Baseline and one-year follow-up pulmonary function tests (PFTs) of all patients. FEV1, forced expiratory volume in 1 s. Figure S3. Baseline and one-year follow-up pulmonary function tests (PFTs) of all patients. DLCO, diffusion capacity for carbon monoxide. Table S2. Baseline and one-year follow-up pulmonary function tests (PFTs) of patients undergoing anti-inflammatory treatment. FVC, Forced Vital Capacity; FEV1, forced expiratory volume in 1 s; DLCS, diffusion capacity for carbon monoxide. Figure S4. Baseline and one-year follow-up pulmonary function tests (PFTs) of patients undergoing anti-inflammatory treatment. FVC, Forced Vital Capacity. Figure S5. Baseline and one-year follow-up pulmonary function tests (PFTs) of patients undergoing anti-inflammatory treatment. FEV1, forced expiratory volume in 1 s. Figure S6. Baseline and one-year follow-up pulmonary function tests (PFTs) of patients undergoing anti-inflammatory treatment. DLCO, diffusion capacity for carbon monoxide.
