# Supplementary material for: Functional Improvement at One Year in Fibrotic Interstitial Lung Diseases—Prognostic Value of Baseline Biomarkers and Anti-Inflammatory Therapies
Source: Diagnostics (Basel). 2024 Jul 17;14(14):1544. doi: 10.3390/diagnostics14141544 (PMC11275397; doi:10.3390/diagnostics14141544)
Supplement: Supplementary file 1 [file diagnostics-14-01544-s001.zip › diagnostics-3079561-supplementary.pdf]

Table S1. Baseline and one-year follow-up pulmonary function tests (PFTs) of all patients. FVC, Forced Vital Capacity; FEV1, forced expiratory volume in 1 second; DLCS, diffusion capacity for carbon monoxide

Mean values, n = 142, progredient (73), stable (25), improvement (44)

		FVC (Liter)	FEV1 (Liter)	DLCO (mmol/(min*kPa))
PROGRESSION	Baseline	3.0 (0.9)	2.4 (0.7)	4.5 (1.6)
	1 year	2.7 (0.9)	2.2 (0.6)	3.8 (1.8)
	Δ PFTs	−0.3 (0.5)	−0.2 (0.4)	−0.7 (0.9)
STABLE	Baseline	3.3 (1.2)	2.6 (1.0)	5.1 (2.1)
	1 year	3.3 (1.2)	2.6 (1.0)	5.1 (2.1)
	Δ PFTs	0.0 (0.1)	0.0 (0.2)	0.0 (0.3)
IMPROVEMENT	Baseline	2.7 (0.9)	2.1 (0.7)	4.1 (1.6)
	1 year	3.0 (1.0)	2.3 (0.7)	5.0 (2.0)
	Δ PFTs	0.3 (0.4)	0.2 (0.3)	0.9 (1.3)

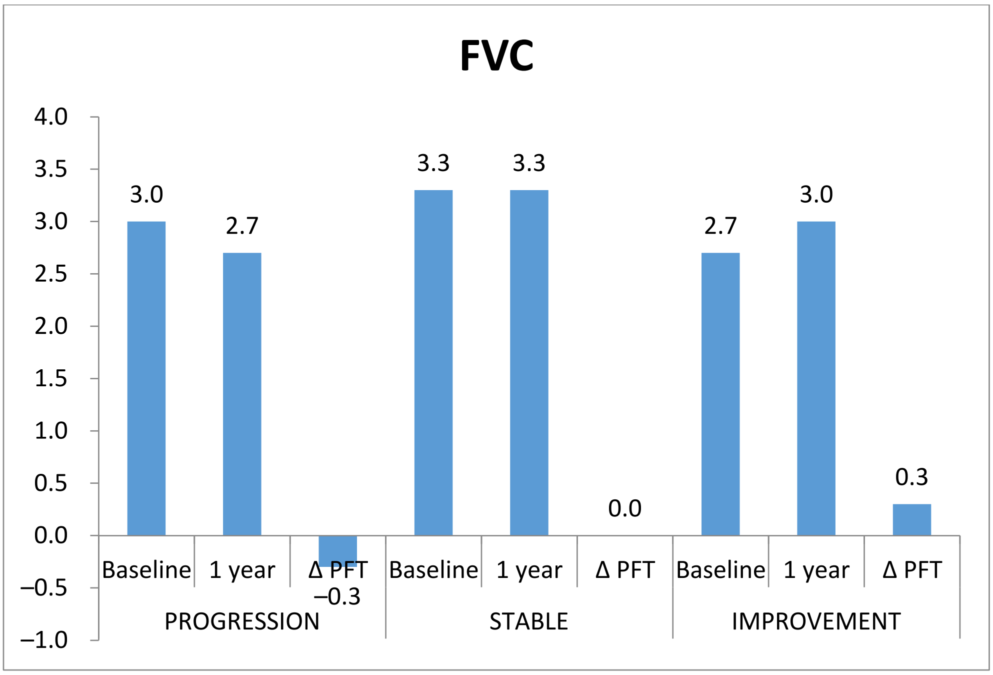

Figure S1. Baseline and one-year follow-up pulmonary function tests (PFTs) of all patients. FVC, Forced Vital Capacity.

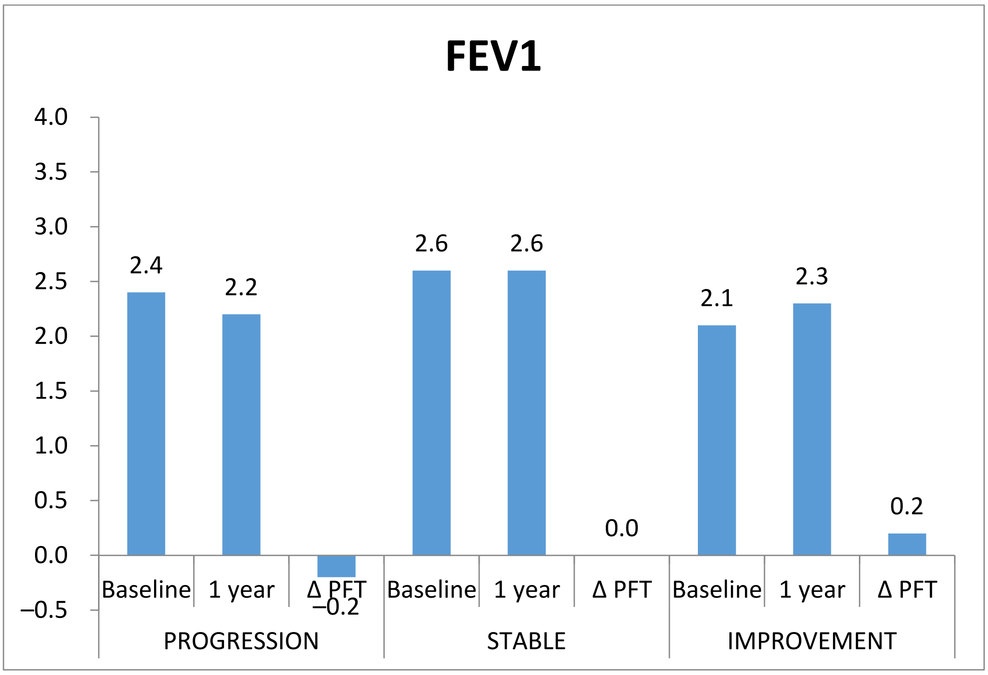

Figure S2. Baseline and one-year follow-up pulmonary function tests (PFTs) of all patients. FEV1, forced expiratory volume in 1 second.

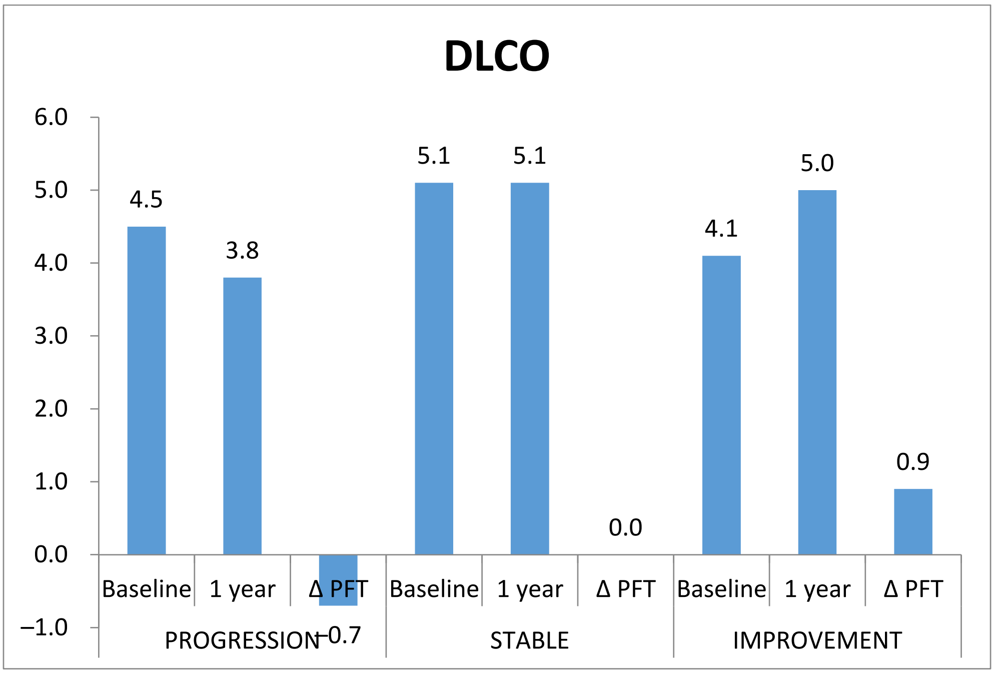

Figure S3. Baseline and one-year follow-up pulmonary function tests (PFTs) of all patients. DLCO, diffusion capacity for carbon monoxide.

Table S2. Baseline and one-year follow-up pulmonary function tests (PFTs) of patients undergoing anti-inflammatory treatment. FVC, Forced Vital Capacity; FEV1, forced expiratory volume in 1 second; DLCS, diffusion capacity for carbon monoxide.

Mean values, n = 84, progredient (38), stable (15), improvement (31)

		FVC (Liter)	FEV1 (Liter)	DLCO (mmol/(min*kPa))
PROGRESSION	Baseline	2.8 (0.8)	2.2 (0.6)	4.2 (1.1)
	1 year	2.6 (0.9)	2.1 (0.6)	3.5 (1.5)
	Δ PFTs	0.2 (0.4)	−0.1 (0.3)	−0.7 (1.0)
STABLE	Baseline	3.6 (1.3)	2.8 (1.0)	5.2 (2.4)
	1 year	3.5 (1.3)	2.8 (1.0)	5.2 (2.6)
	Δ PFTs	0.1 (0.1)	0.0 (0.2)	0.0 (0.4)
IMPROVEMENT	Baseline	2.6 (0.9)	2.2 (0.7)	4.1 (1.6)
	1 year	3.0 (1.0)	2.3 (0.8)	5.1 (2.2)
	Δ PFTs	0.4 (0.4)	0.2 (0.3)	1.0 (1.4)

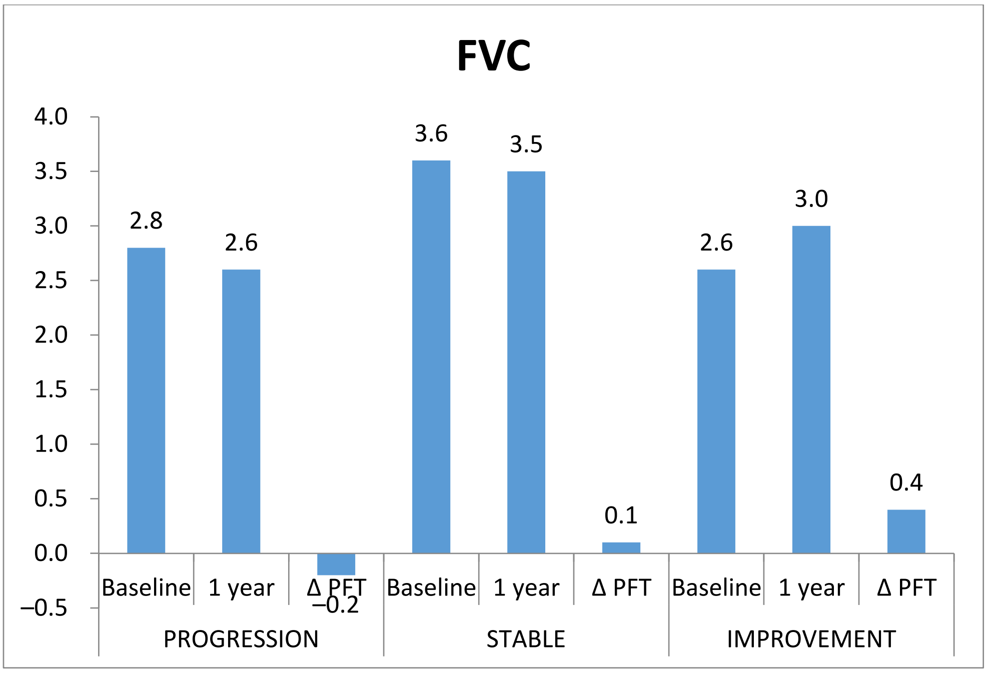

Figure S4. Baseline and one-year follow-up pulmonary function tests (PFTs) of patients undergoing anti-inflammatory treatment. FVC, Forced Vital Capacity.

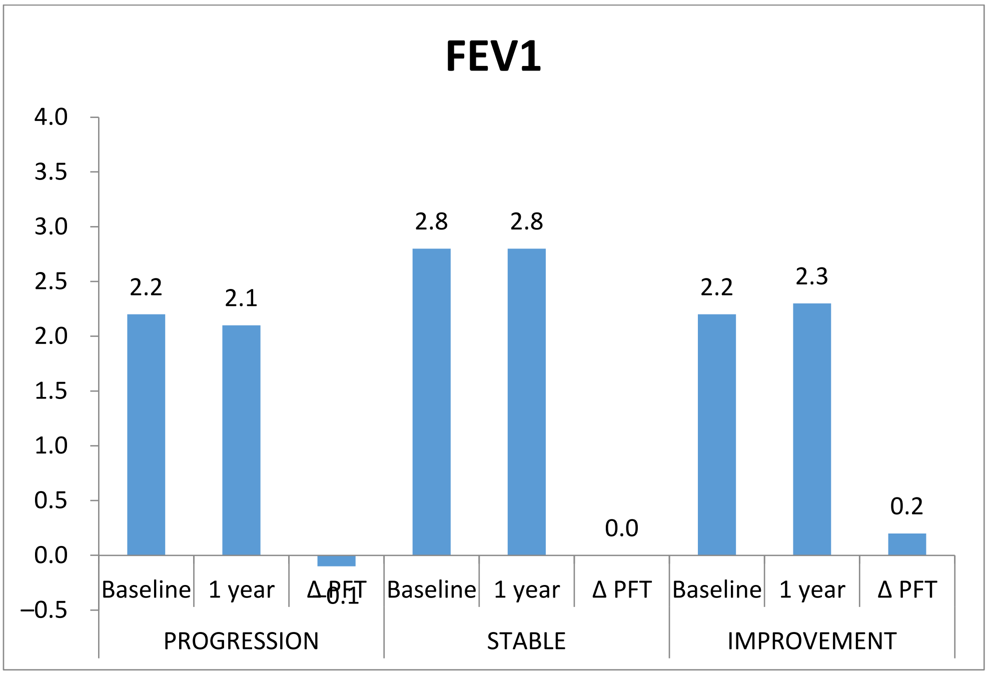

Figure S5. Baseline and one-year follow-up pulmonary function tests (PFTs) of patients undergoing anti-inflammatory treatment. FEV1, forced expiratory volume in 1 second.

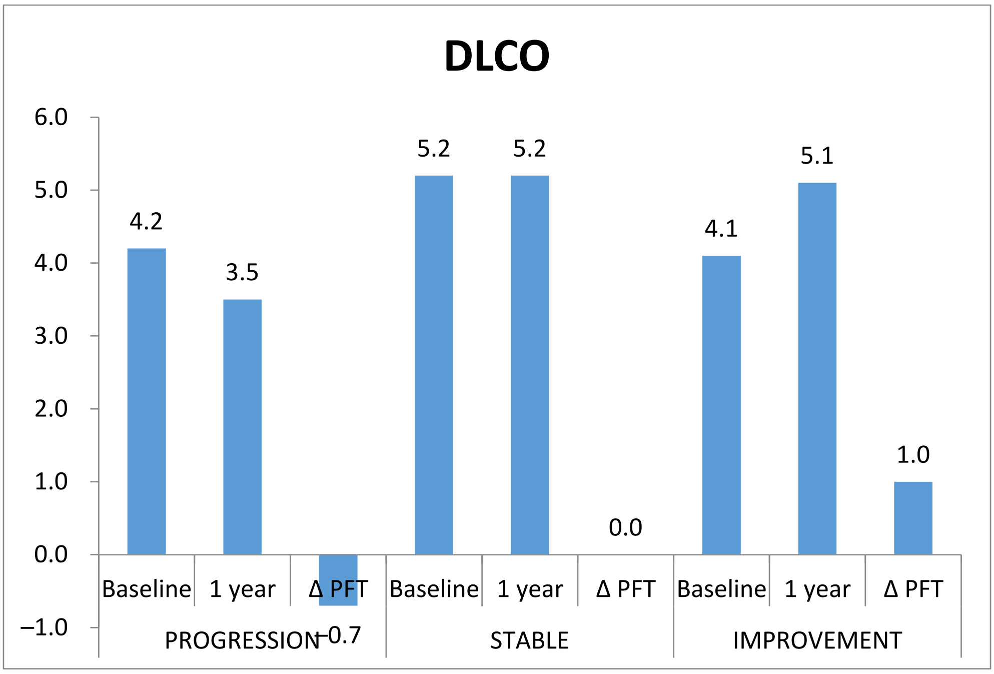

Figure S6. Baseline and one-year follow-up pulmonary function tests (PFTs) of patients undergoing anti-inflammatory treatment. DLCO, diffusion capacity for carbon monoxide.
